# The role of optical coherence tomography angiography in assessing diabetic choroidopathy: a systematic review

**DOI:** 10.1186/s40942-024-00618-5

**Published:** 2025-01-31

**Authors:** M. Hossein Nowroozzadeh, Mansoureh Bagheri

**Affiliations:** 1 Poostchi Ophthalmology Research Center, Department of Ophthalmology, School of Medicine, Shiraz University of Medical Sciences, Shiraz, Iran; 2 Department of Surgical Subspecialties, Service of Ophthalmology at Health Sciences North, Northern Ontario School of Medicine University, Sudbury, ON P3E 5J1 Canada

**Keywords:** Choriocapillaris, Choroidal vasculature, Diabetic choroidopathy, Diabetic retinopathy, Optical coherence tomography angiography

## Abstract

**Background:**

Diabetic retinopathy (DR) is a leading cause of vision impairment worldwide, affecting both retinal and choroidal vasculature. While advances in imaging technology, particularly optical coherence tomography angiography (OCTA), provide new opportunities to assess choroidal changes in diabetic patients, the role of OCTA in early diagnosis and monitoring of diabetic choroidopathy remains unclear.

**Objective:**

This review aims to evaluate the potential role of OCTA in diagnosing and monitoring diabetic choroidopathy.

**Methods:**

A systematic review was conducted following Preferred Reporting Items for Systematic Reviews and Meta-Analyses (PRISMA) 2020 guidelines. Databases including PubMed, Embase, Cochrane Library, Google Scholar, ISI, and Scopus were searched for studies on diabetic choroidopathy assessed by OCTA. Studies included were peer-reviewed, published in English, and excluded case reports, conference proceedings, and studies on treated DR patients. Two independent reviewers screened articles for eligibility based on predefined criteria.

**Results:**

OCTA allows for non-invasive, high-resolution visualization of retinal and choroidal microvasculature, providing both qualitative and quantitative data. The majority of studies indicate a significant decrease in choroidal perfusion parameters in diabetic patients without DR compared to healthy controls. Conflicting evidence exists regarding the correlation between choriocapillaris flow reduction and DR severity. OCTA may also predict changes in visual function related to choroidal perfusion, though it cannot fully replace clinical examinations.

**Conclusions:**

OCTA is a valuable tool for early detection and monitoring of diabetic choroidopathy. However, its role is limited by variability in findings and its inability to detect certain features of diabetic microangiopathy. Further studies are needed to clarify its clinical utility and standardize assessment methods.

**Supplementary Information:**

The online version contains supplementary material available at 10.1186/s40942-024-00618-5.

## Background

Diabetic chorioretinopathy, a leading cause of acquired vision impairment worldwide, affects both the retinal and choroidal vasculature [1]. Histopathologic studies of diabetic choroidopathy have demonstrated arteriosclerotic changes in choroidal vessels and alterations in the choriocapillaris, including capillary dropout, which lead to ischemic changes in the outer retinal layers [2, 3]. Diabetes may also cause basement membrane thickening around the choriocapillaris, microaneurysm formation, and narrowing or even obstruction of the choroidal vasculature, all contributing to macular dysfunction [2, 4]. However, choroidal imaging in diabetic patients, particularly in the early stages of diabetic retinopathy (DR), remains underutilized due to the inherent challenges of visualizing the choroid in vivo [1, 5].

Historically, imaging modalities such as Indocyanine green angiography (ICGA) and Fluorescein angiography have been employed to detect vascular changes in diabetic choroidopathy [5]. Despite their utility, these methods are considered invasive and may not be ideal for visualizing the choriocapillaris due to their limited capacity for depth resolution and lateral imaging [5]. For instance, although selective choriocapillaris filling on ICGA has been suggested as a marker of early diabetic choroidopathy, it lacks the resolution and depth-resolved imaging needed for precise quantification of choriocapillaris flow [6].

Advances in imaging technology, particularly optical coherence tomography angiography (OCTA), offer new possibilities for investigating the pathophysiologic effects of diabetes on the choroidal vasculature in vivo [7]. OCTA utilizes motion contrast imaging to detect moving erythrocytes, causing decorrelation of pixels in sequential optical coherence tomography (OCT) B-scans, which correlates with blood flow velocity [5]. OCTA indices have demonstrated high precision in identifying microvascular changes in both the retinal and choroidal regions, often before detectable fundus or clinical signs appear [5, 8]. Previous studies have reported a significant reduction in choroidal vascular perfusion in diabetic patients compared to healthy controls [9, 10]. This review article aims to evaluate and highlight the potential role of OCTA in the diagnosis and monitoring of diabetic choroidopathy.

## Methods

This systematic review was conducted following the guidelines set by the Preferred Reporting Items for Systematic Reviews and Meta-Analyses (PRISMA) 2020 statement [11]. Two independent reviewers performed a comprehensive search across multiple databases, including PubMed, Embase, the Cochrane Library Central Register of Controlled Trials, Google Scholar, ISI, and Scopus. The most recent search was conducted on September 23, 2024. The search strategy included the terms “OCTA” OR “OCT angiography” OR “optical coherence tomography angiography” AND “diabetes” OR “diabetes mellitus” OR “diabetic choroidopathy” OR “diabetic vasculopathy” OR “diabetic angiopathy”, incorporating both MeSH terms and relevant synonyms.

The review included studies that specifically investigated alterations in choroidal vasculature attributed to diabetes using OCTA. Only articles published in English in peer-reviewed journals were considered, while case reports, abstracts, conference proceedings, letters, and duplicated studies were excluded. No restrictions were placed on age, type of diabetes or its control, or follow-up duration. Studies involving OCTA findings in diabetic patients who had received treatment for DR, including laser therapy or intraocular injections, were also excluded.

**Fig. 1:**
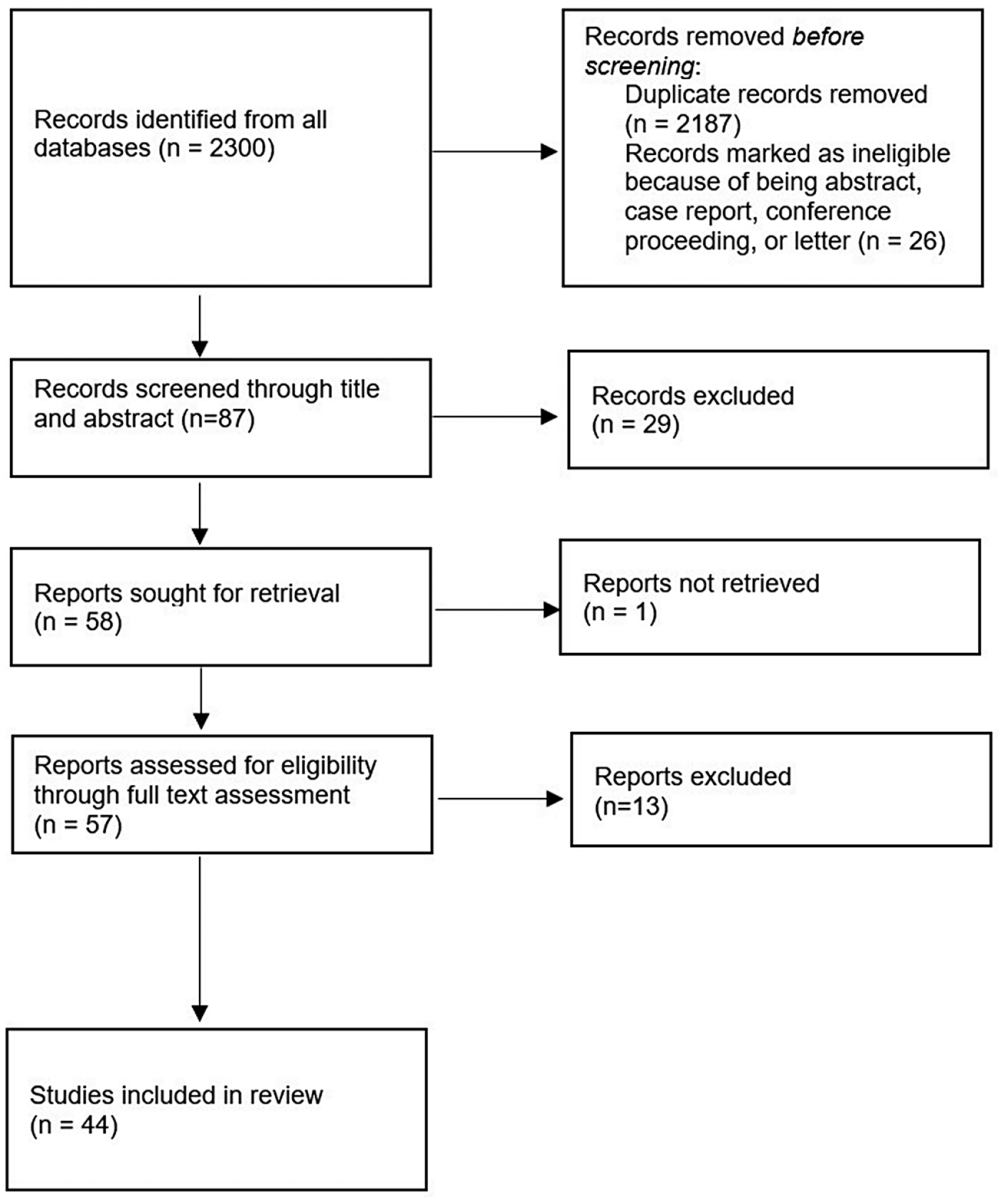
Systematic review flow diagram on the role of optical coherence tomography angiography in assessing diabetic choroidopathy

Titles and abstracts of all retrieved articles were screened independently by two reviewers to identify those meeting the predefined inclusion and exclusion criteria. Articles selected from this initial screening underwent a second, more detailed evaluation of their full texts to confirm compliance with the eligibility criteria (Fig. 1).

For quality assessment, we employed the Newcastle-Ottawa Scale for evaluating cohort and case-control studies. The Newcastle-Ottawa Scale is a star-based rating system that assesses studies across three broad categories: exposure, comparability, and outcome, with a maximum score of nine stars [12].

## Results

Tables 1 and 2 summarize the characteristics and findings of previous cohort, cross-sectional and case-control studies on the role of OCTA in assessing diabetic choroidopathy.

**Table 1:** Summary of cohort studies on the role of OCTA in assessing diabetic choroidopathy

Study	Newcastle-Ottawa Quality Assessment scale	Device	Population Sample size	Outcome measures	Findings
Fragiotta et al. 2023 [28]	6	SS-OCTA	-T1DM with mild NPDR: 22 cases (22 eyes)	-OCTA: CC FD % -AO: CD, LDi, and HPi -Fu: 4 y	- The CC FD exhibited a consistent increase over time (*P* < 0.01). - The LDi and HPi were primarily impacted by choriocapillaris perfusion in the parafovea (*P* = 0.02). - There was progressive deterioration of cone metrics over time due to worsening CC perfusion deficit.
Chen et al. 2023 [44]	6	SS-OCTA	-T2DM without DR or with mild NPDR (no DME): 903 cases	-CC FD% -≥2 steps DR progression or development of DME -Fu: 3 y	− 16.34% developed DR progression, and 6.54% developed DME. - A higher average CC FD% was correlated with DR progression (OR, 3.41 per SD increase, 95% CI: 2.65–4.39, *P* < 0.001) and DME development (OR, 1.37 per SD increase, 95% CI: 1.06–1.77, *P* = 0.016) (especially in the inferior field) after adjusting for confounders.
Guo et al. 2023 [45]	6	SS-OCTA	-T2DM without DR or with mild NPDR (no DME): 946 cases (1879 eyes)	-Parapapillary (3-mm) CC FD% -≥2 steps DR progression or development of DME -Fu: 3 y	− 16.60% experienced DR progression and 6.12% developed DME. - The DR progression was related to an elevated CC FD% (1.62, 1.40–1.88; *P* < 0.001). - The DME occurrence was associated with a higher CC FD% (1.29, 1.03–1.61; *P* < 0.001).
Wang et al. 2022 [40]	4	SS-OCTA	-T2DM: 1222 patients (1222 eyes)	-CC FD% -1-year incidence of RDR	- Each 1% increase in baseline CC FD% was associated with 1.69 times (RR 2.69; 95% CI 1.53 to 4.71; *p* = 0.001) higher risk for development of RDR after 1 year.

AO, adaptive optics; CC, choriocapillaris; CD, cone density; CCFV, choriocapillaris flow void; DR, diabetic retinopathy; DME, diabetic macular edema; FD, flow deficit; FU, follow-up; HPi, heterogeneity packing index; LDi, linear dispersion index; NPDR, non-proliferative diabetic retinopathy; OCTA, optical coherence tomography angiography; OR, odds ratio; RDR, proliferative diabetic retinopathy; RR, relative risk; SD, standard deviation; SS-OCTA, swept-source optical coherence tomography angiography; T1DM, type 1 diabetes mellitus; T2DM, type 2 diabetes mellitus

**Table 2:** Summary of cross-sectional and case-control studies on the role of OCTA in assessing diabetic choroidopathy

Study (Quality)	Newcastle-Ottawa Quality Assessment scale	Device	Population Sample size	Outcome measures	Findings
Thaker et al. 2023 [29]	N/A	SS-OCTA	NPDR: 110 patients (176 eyes)	-Choroid circulation flow void	- Choroid circulation flow void was significantly associated with more severe NPDR.
Bandello et al. 2023 [50]	9		-Total: 60 cases -Treatment-naïve mild DR (ETDRS 20–35): 30 -HC: 30		The CC FD% was associated with foveal mesopic sensitivity (β: -0.234, *p* = 0.046), parafoveal mesopic sensitivity (β: -0.312, *p* = 0.032), and parafoveal dark-adapted sensitivity (β: -0.282, *p* = 0.048).
Deng et al. 2023 [31]	9		-T2DM (not severe DR, no DME): 102 cases (194 eyes) -HC: 28 cases (56 eyes)	-CCF -ERG	- CCF was decreased from the control to the nondiabetic retinopathy (NDR) to DR group. In patients with diabetes, CCF was correlated with ERG parameters (coefficient index=-0.601, *P* < 0.001 with 16 Td-s implicit time; coefficient index=-0.687, *P* < 0.001 with 32 Td-s implicit time; coefficient index = 0.933, *P* = 0.035 with 32 Td-s amplitude). CCF was correlated with the RPE thickness and the level of HbA1c (both *P* = 0.001).
Wang et al. 2023 [30]	N/A	SS-OCTA	-T2DM: 1692 cases	-CC FD density -CC FD size -CC FD number	- After controlling for confounding factors, individuals with DR had a significantly higher FD density compared to those without DR. Specifically, the differences were 1.61% (95% CI 1.04 to 2.18; *p* < 0.001) for individuals with mild NPDR, 2.23% (95% CI 1.76 to 2.70; *p* < 0.001) for those with moderate NPDR, and 3.31% (95% CI 2.27 to 4.36; *p* < 0.001) for those with severe NPDR, compared to individuals without DR. - The higher FD number and size were correlated with more severer degrees of DR (all *p* < 0.05). - Adding FD density to conventional risk factors significantly improved the ability to distinguish DR from NDR patients, with an AUC of 0.829 (95% CI 0.804 to 0.855; *p* < 0.001).
Viggiano et al. 2023 [52]	9	SS-OCTA	-T1DM (*n* = 40) including: NDR: 12, and mild NPDR: 28 -HC: 10	-OCTA: CC FD% -AO: CD, LDi, HPi	- A close relationship was showed between cone metrics and CC FD in NPDR group. - The percentage increase in CC FD was linked to an increase in LDi (*p* = 0.035). Moreover, the rise in CC FD% was found to be correlated with a decrease in CD (*p* = 0.042) and HPi (*p* = 0.017).
Tan et al. 2023[20]	8		-HC: 27 cases (35 eyes) -DM: 75 cases (132 eyes), including: NDR: 62 eyes NPDR: 51 eyes, and PDR: 19 eyes	-CC flow void density, % (3*3 mm; fovea-centered)	- CC parameters were strongly altered with DR stages (*p* < 0.01). - The CC parameters were more effective in distinguishing between the group with no DM & NDR, compared to the retinal parameters (with AUC of 0.954 vs. 0.821, *p* = 0.006). - Combining both retinal and choroidal microvasculature parameters in a classification model resulted in a significant enhancement in distinguishing between DR and the absence of DR, compared to using each parameter independently (*p* = 0.029).
Xiong et al. 2022 [17]	9	SS-OCTA	-DM: 71 cases -High myopia: 71 -DM + High myopia: 71 -HC: 71	-CC perfusion index	The mean CC perfusion index for the control, diabetes, high myopia, and diabetes with high myopia groups were 91.11 ± 0.84%, 90.16 ± 1.46%, 89.80 ± 1.42%, and 89.36 ± 1.19%, respectively (*P* < 0.001).
Kaderil et al. 2022 [49]	N/A	SD-OCTA	-Total: 58 patients (95 eyes), including: 1- NPDR: 59 2- PDR: 36 (2 A: VA ≤ 0.2 logMAR; 2B: > 0.2)	-CC plexus flow area	- CCP flow area was lower in group 2B (All, *p* < 0.05).
Liu et al. 2022 [41]	7	SS-OCTA	-NPDR: 42 cases (71 eyes) -PDR: 31 cases (53 eyes) -HC: 30 cases (51 eyes)	-choroid perfusion	- The choroid perfusion exhibited significant differences across different areas and amongst the three groups.
Zhang et al.,2021 [10]	7		-36 diabetic patients (54 eyes), including: NDR: 31 eyes, Mild NPDR: 19 eyes, and Moderate NPDR: 4 eyes -32 control subjects (54 healthy eyes)	-CC blood flow signal density	Within the diabetic group, the CC blood flow signal density in the macular area (diameter = 2000 μm) exhibited a significant reduction compared to the healthy control group (*P* < 0.05).
Ryu et al. 2021 [39]	7		-DM NDR: 49 -NPDR: 51 -PDR: 38 -HC: 52	-CC vessel density	In NDR group, CC vessel density was found to be decreased only in the perifoveal area (with p-values of 0.823 for the foveal area, 0.631 for the parafoveal area [1–3 mm], and 0.039 for the perifoveal area [3–6 mm]). Multivariate linear regression analyses showed significant associations between DR severity and all retinal and choroidal microvascular indices.
Zlatanović et al. 2021 [16]	8	SD-OCTA	-T2DM without DR: 83 cases (166 eyes); age: 59 ± 14 y -HC: 33 cases (66 eyes)	-CC flow area and vessel density	- There was a statistically significant reduction in the CC flow area and vessel density in the NDR group compared to healthy subjects.
Parravano et al. 2021 [53]	N/A		-T1DM, including: NDR: 18 NPDR: 25	-EZ “normalized” reflectivity, -mfERG rRADs -FDn, FDa, & FD%	-The mean values for EZ “normalized” reflectivity, mfERG RAD, CC FDn, and FD% were not significantly different between the NPDR and NDR groups (*p* > 0.05), but FDa was significantly higher in NPDR eyes (*p* < 0.05). -In NPDR eyes, there was a significant linear relationship between decreased mfERG RADs and increase in either CC FDa and FD%. -In NDR eyes, EZ “normalized” reflectivity was negatively correlated with CC FD%.
Stulova et al. 2021 [19]	7		-T1DM NDR: 41 patients (76 eyes) -HC: 31 (55 eyes)	-CC FDs	-Patients with T1DM showed an increase in both the density and mean size of FDs.
Ra et al. 2021 [42]	N/A		-T2DM: 152 cases (282 eyes), including: NDR: 114 NPDR: 79 S-NPDR: 48 PDR: 41	-CC vascular density -Choroidal vascular density	-CC vascular density was lower in patients with PDR compared to those with NPDR (*P* < 0.05). -Significant positive correlations were found between superficial and deep retinal vascular density and CC vascular density (all *P* < 0.001), while choroidal vascular density showed negative correlations with these parameters (also *P* < 0.001). -Both retinal and CC vascular density were negatively correlated with diabetic retinopathy grade (all *P* < 0.001), while choroidal vascular density had a weak positive correlation (*P* = 0.030).
Ucgul et al. 2021 [55]	N/A		-T2DM, including: NDR without MA: 20, NDR + MA: 20, and Mild NPDR + MA:30	- CC vascular density	There were significant inverse correlations between diabetes duration, creatinine, urea, serum Na, and certain CC vessel density values (with p-values below 0.05 for all correlations).
Ghassemi et al. 2021 [43]	N/A		-DR 97 cases (188 eyes)	-CC vascular density (macula 3*3 mm)	- There was a consistent pattern of decreasing CC vascular density from normal cases to cases of NDR and NPDR, followed by a slight increase in the PDR stage, but the vascular density never reached normal levels. - BCVA was positively correlated with CC vascular density of foveal area.
Agra et al. 2021 [27]	8		-T2DM NDR: 30 cases (30 eyes) -HC: 30 cases (30 eyes)	-CC flow area	-Patients with diabetes showed a slight increase in CC flow area compared to controls (mean area of 22.6 ± 3.9 mm2 vs. 22.3 ± 4.6 mm2, *p* = 0.017). -There was a positive correlation between fasting blood glucose levels and CC flow area (*p* = 0.034).
Loria et al. 2021 [15]	7	SS-OCTA	-HC: 17 -NDR: 30 -Minimal NPDR:22 -Moderate NPDR:30 -Severe NPDR:16 -PDR: 5	-CC Flow void area	-There was a significant positive correlation between CC flow void area and DR stage. Furthermore, FVA-CC was significantly higher in diabetic patients without DR compared to healthy individuals (*P* = 0.008).
Ro-Mase et al. 2020[51]	8	SD-OCT	-T2DM: 26 cases including: NDR: 4, NPDR:12, and PDR:10 -HC: 13	-CC FD -Microperimetry: Retinal sensitivity -AO: HPi	-In patients with NPDR and PDR, there was a significant correlation between foveal and CC FD and retinal sensitivity (fovea, *r* = -0.58; *P* = 0.046 and *r* = -0.82; *P* = 0.003; parafovea, *r* = -0.59; *P* = 0.044 and *r* = -0.72; *P* = 0.019, respectively), but not in control and NDR groups. -No significant differences were found in HPi across all groups.
Dai et al. 2020 [1]	8	SS-OCTA	-DM: 45 with DR -HC: 27 -Extreme axial lengths of *>* 25.2 mm or *<* 23.2 mm were excluded	-CC FD (5-mm from 6*6 mm scans)	- Compared to control eyes, diabetic eyes had a significantly higher CC FD% (12.34 ± 4.14% vs. 8.82 ± 2.61%, *P* < 0.001) and larger mean CC FD size (2151.3 ± 650.8µm2 vs. 1574.4 ± 255.0 µm2, *P* < 0.001), with both values being 1.4-fold greater on average. - There was no significant difference in CC FD% or mean CC FD size between eyes with NPDR and those with PDR (*P* = 1.000 and *P* = 1.000, respectively).
Saif et al. 2020 [9]	7	SS-OCTA	-HC: 16 eyes -NDR: 16 eyes -NPDR: 16 eyes -PDR: 16 eyes	-Choroidal vascular density	-There was a decrease in choroidal vascular density in diabetic patients compared to healthy control group.
Ashraf et al. 2020[35]	N/A	3*3 mm macular scan	-225 cases Type 1 or Type 2 DM (352 eyes), including: Mild NPDR:183, Moderate NPDR:71, Sever NPDR or PDR:98	-CC FD	Among eyes without predominantly peripheral lesions, the mean CC flow density decreased with increasing DR severity (mild NPDR, 69.7% [6.2%]; moderate NPDR, 67.6% [5.6%]; severe NPDR or PDR, 67.1% [5.6%]; *P* = 0.01). -However, in eyes with predominantly peripheral lesions, the mean CC flow density did not appear to change with increasing DR severity (mild NPDR, 67.1% [5.6%]; moderate NPDR, 69.3% [4.6%]; severe NPDR or PDR, 68.3% [5.6%]; *P* = 0.49).
Dai et al. 2020 [6]	8	SS-OCTA	-NDR: 16 cases (16 eyes) -HC: 16	-CC FD% -CC FD size	-In diabetic eyes, compared to controls, there were significant increases in mean FD% and mean FD sizes in the central 1.0-mm disk (*P* = 0.011 and *P* = 0.017), the central 1.5-mm rim (*P* = 0.003 and *P* = 0.009), the central 2.5-mm rim (*P* = 0.018 and *P* = 0.020), and the entire 5.0-mm disk (*P* = 0.009 and *P* = 0.008). -There were no significant differences in any retinal vessel quantitative parameters between the two groups (all *P* > 0.05).
Gendelman et al. 2020 [7]	N/A	SS-OCTA	-90 DM cases (160 eyes), including: NDR: 33, Mild NPDR:17, Moderate NPDR: 8, Severe NPDR: 10, and PDR:22	-CC FD	-Age and severity of DR were significantly and positively associated with FD% in all studied regions, with a greater effect observed in the two centermost regions. -The increase in FD percentage per year of age varied by region: 0.12 for the inner (*p* < 0.001), 0.09 for the middle (*p* < 0.001), 0.05 for the outer (*p* < 0.001), and 0.06 for the full-field (*p* < 0.001). -The increase in FD percentage per increase in DR severity stage also varied by region: 0.65 for the inner (*p* < 0.0087), 0.56 for the middle (*p* < 0.0012), 0.33 for the outer (*p* < 0.045), and 0.36 for the full-field (*p* < 0.018).
Lupidi et al. 2020 [37]	7		-29 diabetic patients with Level 20 DR severity score -HC: 20	-CC vascular perfusion density -SCP and DCP vascular perfusion density	In diabetic patients, vascular perfusion density values were significantly lower in the DCP (25.1% vs. 26.5%; *p* = 0.04) and CC (71.2% vs. 86.6%; *p* = 0.0001) compared to controls. -A negative linear correlation was found between CC vascular perfusion density and DCP vascular perfusion density in diabetic patients, while a positive linear correlation was observed between the same parameters in controls.
Borrelli et al. 2020 [54]	7		-NPDR: 30 -HC: 30	-EZ normalized reflectivity -CC perfusion density	-The NPDR group had impaired perfusion in both retinal and choroidal vasculature, and a lower “normalized” reflectivity compared to controls (0.73 ± 0.19 vs. 0.96 ± 0.25, *P* < 0.0001). -Multiple regression analysis showed a significant direct association between EZ “normalized” reflectivity and CC perfusion density in NPDR patients (*P* = 0.025) but not in controls (*P* = 0.476).
Forte et al. 2020 [14]	7	SS-OCTA	-T1DM NDR: 17 -T2DM NDR: 17 -23 HC	-CC voids	-There was a higher frequency of CC voids in both DM Type 1 and DM Type 2 compared to controls (*P* = 0.003 and *P* < 0.001, respectively).
Sacconi et al. 2019 [23]	9	SD-OCTA	-T1DM NDR: 34 cases (34 eyes) -HC: 27	-CC perfusion density	-OCTA showed lower deep capillary plexus perfusion density in diabetics than the control group, but no significant differences were found in other retinal/choriocapillaris plexuses or foveal avascular zone area.
Mastropasqua et al. 2019 [46]	8	SS-OCTA	-94 cases (95 eyes), including: NDR: 25, Mild NPDR: 23, Mod/sever DR: 26, and PDR: 20 -HC: 25	-CC, SCP and DCP perfusion density (central vs. peripheral)	-The prediction of disease worsening had excellent specificity and good sensitivity, particularly in the central and temporal sectors across all plexuses.
Yang et al. 2019 [4]	8		-HC: 43 -NDR: 56 -Mild DR: 43 -Moderate DR: 54 -Severe DR: 38 -PDR: 48	-CC flow density	-As DR progressed, there was a downward trend in CC flow density in the CC layer. -Significant differences were found between mild NPDR and moderate NPDR and between severe NPDR and PDR in the comparison of CFD in the CC layer using both 3-mm and 6-mm scan patterns (*P* = 0.003, *P* = 0.001).
Yang et al. 2019 [38]	8		-HC: 40 -Mild DR: 40 -Moderate DR: 40 -Severe DR: 40 -PDR: 40	-CC vascular density	-As DR severity increased, there was a reduction in macular perfusion in the SCP, DCP, and CC. -Vessel density in the DCP was found to be a better indicator of DR severity, with an area under the curve, sensitivity, and specificity of 0.967, 92.5%, and 93.1%, respectively, compared to vessel density in the SCP and CC.
Conti et al., 2019 [26]	7	SD-OCTA	-HC: 37 eyes -NDR: 31 eyes -NPDR: 41 eyes -PDR: 27 eyes	-CC capillary perfusion density	-Eyes affected by both NPDR and PDR exhibited a significant reduction in choriocapillaris capillary perfusion density when compared to controls. In contrast, diabetic eyes without retinopathy did not display a noteworthy change. -The whole-image capillary perfusion density of the CC declined by 8.3% in NPDR eyes (*p* < 0.01) and by 7.1% in PDR eyes (*p* < 0.01). Additionally, parafoveal capillary perfusion density in the choriocapillaris showed an 8.9% decrease in NPDR eyes (*p* < 0.01) and an 8.2% decrease in PDR eyes (*p* < 0.01).
Rodrigues et al. 2019 [36]	N/A	SD-OCT	-NPDR: 56 cases (101 eyes)	-CC vascular density	-Univariate analysis revealed that higher ETDRS level was associated with several OCTA parameters, including parafoveal SCP density (OR = 0.87, 95% CI 0.76–0.99, *p* = 0.039), parafoveal DCP density (OR = 0.79, 95% CI 0.72–0.87, *p* < 0.001), and CC density (OR = 0.89, 95% CI 0.80–0.99, *p* = 0.036). -However, after adjusting for relevant clinical features, only parafoveal vessel density in the DCP remained a significant predictor of NPDR ETDRS level (OR = 0.54, 95% CI 0.32–0.92, *p* = 0.024).
Li et al. 2019 [48]	8	SD-OCTA	-T-2DM: 97 patients (NDR to PDR) -HC: 48	-Foveal and parafoveal CC vascular density -Foveal flow area in CC plexus	-Compared to controls, diabetic patients had lower flow area in CC plexus and vascular density in all three layers. -In the NDR group, foveal flow area in CC plexus decreased significantly compared to controls. In mild NPDR, parafoveal VD decreased significantly in all three layers compared to NDR, particularly in the temporal and nasal areas. In moderate NPDR, vascular density reduction extended to the inferior area in SCP and DCP compared to mild NPDR. In severe NPDR, there were progressive losses of vascular density in all layers compared to moderate NPDR. In PDR, the superior vascular density in SCP increased significantly compared to severe NPDR.
Li et al. 2018 [18]	7	SD-OCTA	-T2DM NDR: 42 cases (42 eyes) -HC: 40	-CC flow area	-Reduced CC flow area was significantly more obvious in the NDR subjects than in the control subjects (1.94 ± 0.28 versus 2.05 ± 0.11 mm2, *p* = 0.02).
Cao et al. 2018 [56]	7	SD-OCTA	-T2DM NDR: 71 -HC: 67	-CC vessel density	-NDR group showed a significant reduction in average vessel density of SCP, DCP, and CC (*p* < 0.001, *p* < 0.001, and *p* = 0.006, respectively).
Carnevali et al. 2017 [24]	7	SD-OCTA	-T1DM NDR; 25 cases -HC: 25	-CC vessel density	-Comparison of the DCP between diabetic and control eyes showed a significant decrease in vessel density in the diabetic group [0.464 ± 0.016 vs. 0.477 ± 0.014, respectively (*p* = 0.005)]. -However, no significant differences were found in vessel density in the all-retina plexus, SCP, or choriocapillaris.
Dimitrova et al. 2017 [25]	9	SD-OCTA	-DM NDR: 29 -HC: 32	-CC vessel density	-Compared to control subjects, NDR patients had decreased vessel densities in both superficial and deep retina [44.35% ± 13.31% and 31.03% ± 16.33% vs. 51.39% ± 13.05%, *P* = 0.04; and 41.53% ± 14.08% vs. control, *P* < 0.01, respectively]. -They also observed a tendency towards decrease in CC vessel density in diabetic eyes without DR compared to healthy subjects; however, the difference did not reach statistical significance. -There was a significant negative correlation between CC density and diastolic blood pressure in the NDR group (*r* = − 0.42, *P* = 0.02).
Choi et al. 2017 [5]	8	SS-OCTA	-HC: 32 cases (63 eyes) -PDR: 7 (9 eyes) -NPDR: 16 (29 eyes) -DM NDR: 28 (51 eyes)	-CC flow impairment	-CC flow impairment was observed in patients with either PDR or NPDR. Furthermore, CC flow impairment was found in 24 out of 51 diabetic eyes with no DR.

AUC, area under the curve; BCVA, best-corrected visual acuity; CC, choriocapillaris; CD, cone density; DCP, deep capillary plexus; DM, diabetes mellitus; DME, diabetic macular edema; DR, diabetic retinopathy; ERG, electroretinography; ETDRS, Early Treatment Diabetic Retinopathy Study; EZ, ellipsoid zone; FD, flow deficit; FDa, flow deficit area; FDn, flow deficit number; HbA1c, hemoglobin A1c; HC, healthy controls; HPi, heterogeneity packing index; LDi, linear dispersion index; logMAR, logarithm of the minimum angle of resolution; MA, microalbuminuria; mfERG, multifocal electroretinography; N/A, not applicable; NDR, no diabetic retinopathy; NPDR, non-proliferative diabetic retinopathy; OCTA, optical coherence tomography angiography; OR, odds ratio; PDR, proliferative diabetic retinopathy; RAD, response amplitude density; RPE, retinal pigment epithelium; SCP, superficial capillary plexus; SD, standard deviation; SD-OCTA, spectral-domain optical coherence tomography angiography; SS-OCTA, swept-source optical coherence tomography angiography; T1DM, type 1 diabetes mellitus; T2DM, type 2 diabetes mellitus

E-Tables 1 and 2 provide a quality assessment of cohort and case-control studies based on the Newcastle-Ottawa Scale criteria. In the subsequent sections, we categorize and discuss the key findings of these papers.

### The pros and cons of OCTA in the assessment of choroidal microangiopathy

OCTA offers a three-dimensional visualization of the retinal and choroidal microvasculature at multiple depths [5]. It facilitates detailed examination of the foveal avascular zone’s shape and provides assessment of retinal lesions, including areas of nonperfusion, microaneurysms, and neovascularization [13]. OCTA also enables the visualization of choroidal lesions (such as “medusa,” “sea fan,” “glomerular,” and “dead tree” patterns) and choroidal vessel structures [13]. A significant advantage of OCTA is its ability to perform rapid, high-resolution qualitative and quantitative evaluations of retinal and choroidal circulation at different depths and layers [5, 13]. Additionally, the non-invasive nature of OCTA allows for repeated scans during the same visit or subsequent visits, and it can simultaneously provide structural data during OCT imaging [5].

It is important to note that swept-source OCTA (SS-OCTA) is more reliable than spectral-domain OCTA (SD-OCTA) for visualizing and evaluating choroidal vessels [6]. This superiority is due to the longer laser wavelength used in SS-OCTA, which experiences less scattering by the retinal pigment epithelium (RPE), whereas the shorter wavelength of SD-OCTA is more affected by RPE scattering [6].

OCTA has certain limitations; it cannot detect blood-retinal barrier breakdown or vascular leakage, which are visible with Fluorescein angiography [13]. Other challenges include accurate segmentation of the choriocapillaris, the elimination of projection artifacts, and the lack of standardized terminology for consistent data interpretation [13]. Another drawback is OCTA’s reduced ability to visualize intervascular spaces within the choriocapillaris [13]. This issue arises because the distance between individual choriocapillaris (5–20 μm) is smaller than the lateral resolution of the OCT system (15–20 μm), making it difficult to clearly resolve individual choriocapillaris [6]. To address this, researchers use markers such as the percentage and size of flow deficits to evaluate choriocapillaris perfusion in different macular regions [6]. A flow deficit at the choriocapillaris level is detected when blood flow falls below the detectable threshold of OCTA images [1].

### Comparison of OCTA findings in the choriocapillaris of diabetic eyes without diabetic retinopathy versus healthy individuals

Findings from various studies indicate a significant decrease in choroidal perfusion parameters in diabetic patients without diabetic retinopathy (NDR) compared to healthy individuals [6, 14–19]. For example, Forte et al. observed a higher prevalence of choriocapillaris flow voids in the NDR group with both type 1 and type 2 diabetes compared to healthy subjects (*P* = 0.003 and *P* < 0.001, respectively), with no statistically significant difference between the two diabetic subgroups (*P* = 0.8) [14]. Similarly, Dai et al. reported a significant increase in the percentage and average size of choriocapillaris flow deficits across various regions, including the central 1.0-mm zone (*P* = 0.011 and *P* = 0.017), the central 1.5-mm rim (*P* = 0.003 and *P* = 0.009), the central 2.5-mm rim (*P* = 0.018 and *P* = 0.020), and the entire 5.0-mm zone (*P* = 0.009 and *P* = 0.008) in NDR eyes compared to controls [6]. Additionally, Loria et al. found that diabetic patients without DR had a significantly higher choriocapillaris flow void area than healthy individuals (*P* = 0.008) [15]. Furthermore, Zlatanović et al. demonstrated a significant reduction in choriocapillaris flow area and vascular density in the NDR group compared to healthy subjects (*P* < 0.001 for both) [16].

Another choroidal perfusion parameter, the choroid vascularity index (CVI), is defined as the proportion of the choroidal vascular luminal area to the total choroid area. Previous studies have shown a decrease in CVI in diabetic patients compared to healthy controls [20, 22]. For example, Xu et al. reported a significant decrease in CVI in the choriocapillaris of patients with pre-DR and early-stage DR due to type 2 diabetes, particularly in the more peripheral 9–12 mm area (*P* < 0.05) [21]. They also observed a significant negative correlation between the duration of diabetes and CVI, with the strongest correlation in the 9–12 mm area [21]. Aksoy et al. similarly reported a negative correlation between CVI and diabetes duration in NDR eyes (coefficient: -0.416, *P* = 0.006), suggesting progressive subclinical dysfunction in the choroid of diabetic patients [22].

Tan et al. found that choriocapillaris parameters were more effective in distinguishing NDR from healthy controls compared to retinal parameters (area under the curve of 0.954 vs. 0.821, *P* = 0.006) [20]. However, some authors suggest that alterations in perfusion within the deep capillary plexus (DCP) may serve as a superior marker for early diabetic microangiopathy compared to changes in the choriocapillaris [23]. Sacconi et al. found reduced perfusion density in the DCP layer in the NDR group compared to controls, with no significant differences in other perfusion density measurements in the choriocapillaris or superficial capillary plexus (SCP) [23]. Similarly, Carnevali et al. reported a significant reduction in vessel density in the DCP in NDR eyes compared to controls (*P* = 0.005), while no significant differences were observed in the all-retina plexus, SCP, or choriocapillaris between the groups [24].

While reduced choriocapillaris flow may serve as an imaging marker for detecting early microvascular ischemia in patients with NDR, this reduction in choroid perfusion does not always achieve statistical significance [25]. For instance, Dimitrova et al. reported that although there was a trend toward decreased choriocapillaris vessel density in NDR eyes compared to healthy subjects, this difference did not reach statistical significance [25].

On the other hand, some studies debated the role of OCTA in detecting preclinical alterations in diabetic choroidal vasculature [23, 24, 26, 27]. For instance, Conti et al. found no significant difference in choriocapillaris capillary perfusion density between the NDR group and healthy controls [26]. Additionally, Agra et al. reported a marginal increase in choriocapillaris flow area among NDR patients compared to controls (*P* = 0.017), suggesting that OCTA may not be the most suitable tool for detecting preclinical changes in diabetic patients [27]. They further emphasized that OCTA should not replace clinical examinations [27].

### Association between DR stage and choriocapillaris OCTA indices

Several studies suggest that diabetes-associated alterations in the choriocapillaris detected by OCTA correlate with the stage of diabetic retinopathy (DR) [15, 28–31]. For example, Loria et al. found a significant positive correlation between the choriocapillaris flow void area and the stage of DR (*P* < 0.0001) [15]. Fragiotta et al. reported a continuous increase in the percentage of choriocapillaris flow deficit over time in eyes with mild non-proliferative DR (NPDR) (*P* < 0.01) [28]. Thaker et al. also noted a positive correlation between the number of choroidal flow voids and the severity of NPDR (*P* < 0.0001) [29]. Gendelman et al. found a significant increase in choriocapillaris flow deficit across inner, middle, outer, and full-field macular regions with worsening DR (*P* < 0.0087, *P* < 0.0012, *P* < 0.045, and *P* < 0.018, respectively) [7].

Similarly, Dodo et al. observed that, as DR severity increased from moderate NPDR to severe NPDR and proliferative DR (PDR), the area of choriocapillaris flow void within the central subfield gradually increased (*P* = 0.032, *P* = 0.009, and *P* = 0.002, respectively) [32]. They proposed two explanations for this correlation: first, that outer retinal ischemia may result from disruption in choroidal blood flow, potentially leading to increased vascular endothelial growth factor expression and DR progression [32, 33]; and second, that capillary dropout in the inner choroid might result from diabetes-induced damage to the RPE and subsequent disturbance in choriocapillaris maintenance [32, 34].

Ashraf et al. found that choriocapillaris flow density decreases with increasing DR severity in patients without predominantly peripheral lesions [35]. However, this association was not observed in patients with predominantly peripheral lesions [35]. They concluded that nonperfusion in diabetic eyes likely exists along a spectrum, ranging from central to peripheral nonperfusion predominance [35].

Rodrigues et al. reported a correlation between higher Early Treatment Diabetic Retinopathy Study (ETDRS) levels and various OCTA parameters, including parafoveal SCP density (*P* = 0.039), parafoveal DCP density (*P* < 0.001), and choriocapillaris density (*P* = 0.036) in patients with NPDR [36]. However, after adjusting for pertinent clinical factors, only parafoveal vessel density in the DCP remained a significant predictor of ETDRS level (*P* = 0.024) [36]. Therefore, they concluded that parafoveal vessel density in the DCP is the most robust parameter associated with ETDRS level [36].

Lupidi et al. demonstrated that diabetic patients with a DR severity score of level 20 exhibited significantly lower vascular perfusion density values in both DCP (25.1% vs. 26.5%; *P* = 0.04) and the choriocapillaris (71.2% vs. 86.6%; *P* = 0.0001) compared to controls [37]. An inverse linear correlation was found between these two parameters in diabetic patients, in contrast to a positive linear correlation observed in controls [37]. This suggests that the retinal and choroidal vascular networks, while distinct, may be functionally interconnected, and variations in perfusion levels could represent a shared compensatory response to ischemic injury [37].

Yang et al. also observed a consistent decrease in macular perfusion across the SCP, DCP, and choriocapillaris with increasing DR severity [38]. However, they found that vessel density in the DCP serves as a superior indicator of DR severity, with an area under the curve of 0.967, sensitivity of 92.5%, and specificity of 93.1% [38]. Ryu et al. similarly reported associations between DR severity and all retinal and choroidal microvascular indices, but highlighted that changes were more pronounced in the retinal capillary plexuses compared to the choriocapillaris [39].

Some studies suggest that reduced choroidal perfusion may increase the risk of developing PDR in patients with NPDR [40, 41]. For instance, Wang et al. reported a 1.69-fold higher risk of PDR development for each 1% increase in baseline choriocapillaris flow deficit percentage after following patients with type 2 diabetes for one year [40]. Liu et al. also found a lower choroidal perfusion rate in the PDR group compared to the NPDR group [41]. Ra et al. reported that patients with PDR exhibited lower choriocapillaris vascular density than those with NPDR (*P* < 0.05) [42]. In contrast, Ghassemi et al. found no significant change in choriocapillaris vessel density from the NPDR to PDR stage [43].

Some previous studies also suggest that an increase in choriocapillaris flow deficit may elevate the risk of developing diabetic macular edema in patients with type 2 diabetes [44, 45]. Yang et al. found significant differences in choriocapillaris flow density between patients with and without diabetic macular edema (*P* < 0.001) [4].

While many studies support a correlation between the stage of DR and alterations in choriocapillaris flow with high specificity and good sensitivity [46], others argue that deterioration of choriocapillaris flow does not always align with DR severity [5]. For example, Choi et al. reported no clear correlation between the presence or severity of retinal microangiopathy and choriocapillaris flow impairment in most diabetic eyes [5]. In addition, Yang et al. found significant differences in choriocapillaris flow density between mild NPDR and moderate NPDR and between severe NPDR and PDR on both 3-mm (*P* = 0.001 and *P* = 0.003, respectively) and 6-mm scan patterns (*P* < 0.001 and *P* = 0.001, respectively), but no significant differences when comparing other adjacent stages [4]. They concluded that choriocapillaris flow density worsening does not entirely follow DR severity [4].

Dai et al. also reported no significant differences in the percentage or mean size of choriocapillaris flow deficit between patients with NPDR and PDR (*P* = 1.000 for both) [1]. They proposed that as diabetes progresses, various complicated microangiopathic changes occur in diabetic choriocapillaris [1]. For example, narrowed choriocapillaris lumina and choriocapillaris dropouts contribute to increase in flow deficit size and percentage while formation of microaneurysms, capillary dilation and vascular loops can lead to decreased flow deficit size and percentage [1]. Moreover, they noted that the current OCTA system’s limited lateral resolution prevents detailed analysis of individual choriocapillaris for subtle morphological changes [1]. This makes it unclear why there is no notable correlation between diabetic choroidopathy and DR severity [1].

### Correlation between choroid perfusion parameters and visual function

OCTA can detect small nonperfusion areas at the choriocapillaris level as areas of flow void [47]. Since the RPE, outer retinal layers, and photoreceptors are mainly nourished by the choroidal vasculature, a flow void at the choriocapillaris level may result in damage and disruption to the outer retinal layers [47]. Consequently, previous studies have investigated the potential link between choroidal vascular plexus perfusion and visual acuity [36, 43, 48]. For instance, Li et al. reported a negative correlation between logMAR best-corrected visual acuity (BCVA) and flow area in the choroidal capillary plexus [48]. This negative correlation was also observed between logMAR BCVA and choroidal vascular density in the foveal and parafoveal areas [48]. Similarly, Ghassemi et al. found a positive correlation between BCVA (decimal) and choriocapillaris vascular density in the foveal area (*r* = 0.233, *P* = 0.0021) [43]. Kaderli et al. also observed a significantly reduced choriocapillaris flow area in PDR patients with poorer vision (visual acuity > 0.2 logMAR) compared to those with better vision and PDR [49].

Several studies have also investigated the potential association between retinal photoreceptor function and choroidal vasculature flow [50, 51]. For example, Bandello et al. identified a significant inverse correlation between the percentage of choriocapillaris flow deficit and mesopic sensitivity in the foveal and parafoveal regions, as well as parafoveal dark-adapted sensitivity (β = -0.234, *P* = 0.046; β = -0.312, *P* = 0.032; and β = -0.282, *P* = 0.048, respectively) [50]. This suggests that macular hypoperfusion may contribute to dysfunction in both rod and cone activities [50]. Ro-Mase et al. also found a significant association between foveal and choriocapillaris flow deficit and retinal sensitivity in individuals with NPDR and PDR [51]. The correlation was significant for both the foveal (*r* = -0.58; *P* = 0.046 and *r* = -0.82; *P* = 0.003, respectively) and parafoveal regions (*r* = -0.59; *P* = 0.044 and *r* = -0.72; *P* = 0.019, respectively), but not in the control and NDR groups [51]. However, they found no significant differences in cone heterogeneity packing index (HPi) across all groups [51].

Viggiano et al. reported a strong association between cone metrics and choriocapillaris flow deficit in individuals with NPDR [52]. Specifically, an increase in choriocapillaris flow deficit percentage was linked to a rise in the linear dispersion index (LDi) (*P* = 0.035), while a higher choriocapillaris flow deficit percentage was associated with a decrease in cone density (*P* = 0.042) and HPi (*P* = 0.017) [52]. In a related study, Fragiotta et al. found that LDi and HPi were primarily affected by choriocapillaris perfusion and flow deficit percentage in the parafoveal area of eyes with mild NPDR (*P* = 0.02) [28]. They also reported a progressive deterioration of these cone metrics over time due to worsening choriocapillaris perfusion deficit [28].

Other studies have explored the relationship between alterations in the choriocapillaris and morpho-functional characteristics of the outer retina in individuals with diabetes [53, 54]. For instance, Parravano et al. quantitatively assessed photoreceptor structural integrity by measuring the reflectivity of the ellipsoid zone (EZ) [53]. They also used the multifocal electroretinogram (mfERG) response amplitude density (RAD) to evaluate the functional responses of the outer retina [53]. Their results indicated that the average values of EZ “normalized” reflectivity, mfERG RAD, choriocapillaris flow deficit number, and percentage did not differ significantly between the NPDR and NDR groups (*P* > 0.05) [53]. However, a negative correlation was found between EZ “normalized” reflectivity and choriocapillaris flow deficit percentage in the NDR group [53]. They also found a significant association between abnormal outer retinal function (reduced mfERG RAD) and choriocapillaris characteristics (increased flow deficit area and percentage) in the NPDR group [53]. They hypothesized that the functional impairment of outer retinal elements in patients with NPDR may be proportionate to the deficit in choriocapillaris vascular supply [53].

Borrelli et al. reported impaired perfusion in both retinal and choroidal vasculature, along with lower EZ “normalized” reflectivity in patients with NPDR compared to controls (*P* < 0.0001) [54]. Multiple regression analysis revealed a significant direct association between EZ “normalized” reflectivity and choriocapillaris perfusion density in NPDR patients (*P* = 0.025), an association not observed in controls (*P* = 0.476) [54]. Additionally, Deng et al. found a significant correlation between choriocapillaris flow and electroretinogram (ERG) parameters [31]. They noted a significant progressive increase in the implicit time of the 30 Hz flicker ERG, from the healthy control group to the NDR group, and further to the DR group (*P* < 0.05) [31].

### Correlation between choroid perfusion parameters and systemic conditions

Several studies have explored the potential association between systemic medical conditions and choriocapillaris perfusion in diabetic patients [4, 25, 27, 55]. For example, Dimitrova et al. reported a significant negative correlation between the density and flow index of the choriocapillaris and diastolic blood pressure in the parafoveal area of patients with type 2 diabetes mellitus and no DR (*r* = -0.42, *P* = 0.02) [25].

Yang et al. used multiple linear regression analyses to investigate the correlation between systemic medical conditions and choriocapillaris flow density [4]. Their results showed a significant correlation between choriocapillaris flow density in the choriocapillaris layer and factors such as HbA1C, coronary artery disease, atherosclerosis in other locations, estimated glomerular filtration rate, and axial length of the eyes [4]. They also found a significant association between dyslipidemia and choriocapillaris flow density in the choriocapillaris layer on 6-mm scan patterns [4].

Additionally, Agra et al. found a positive correlation between fasting blood glucose levels and choriocapillaris flow area (*P* = 0.034) [27]. In another study, Ucgul Atilgan et al. reported significant negative associations among diabetes duration, creatinine, urea, serum sodium, and specific choriocapillaris vessel density values, with all correlations showing p-values below 0.05 [55].

### Powers and limitations

This review provides an extensive evaluation of relevant studies assessing OCTA parameters for diabetic choroidopathy. The systematic approach adopted in this study helps gauge the usefulness of various OCTA indices as diagnostic and prognostic tools in patients with diabetes. We believe that a meta-analysis would be of limited value and potentially misleading due to significant heterogeneity among the studies in terms of methodology, population, devices used, outcome measures, and severity of diabetic retinopathy. The findings of this review should be considered in the context of the target population and should not be generalized to individuals with different demographic or clinical characteristics, or to devices with different specifications.

## Discussion

The findings from several studies indicate a notable reduction in choroidal perfusion parameters during the preclinical stages of diabetes compared to the healthy population [6, 14–19, 56]. However, some studies have claimed that diabetic patients without DR do not exhibit significant variations in choroidal vascular indices [23, 24, 26, 27]. Therefore, while OCTA can aid in the early diagnosis of choroidal microangiopathy, it is essential to emphasize that OCTA should not be used as a substitute for clinical examinations.

In addition, although many studies have demonstrated a stage-sensitive decline in choroidal microvasculature with increasing disease severity, some researchers have questioned the correlation between choroidal vascular alterations and DR stage. This raises the possibility that diabetic choroidopathy may be a consequence of diabetes itself rather than a contributing factor to the pathogenesis of DR [57].

OCTA of the choriocapillaris can also be used to predict changes in visual function. Since the RPE, outer retinal layers, and photoreceptors primarily rely on oxygenation and nourishment from the choroidal vasculature, a reduction in choriocapillaris perfusion could contribute to a decline in visual function. Previous studies have proposed a link between choroidal vasculature flow in diabetic patients and the morpho-functional characteristics of the outer retina and photoreceptors.

It is important to consider the influence of confounding factors and concurrent health conditions that may affect choroidal circulation when evaluating the choroid in diabetic patients. These factors include, but are not limited to, aging, hypertension, nephropathies, dyslipidemia, coronary artery disease, and atherosclerosis in various regions of the body.

## Electronic supplementary material

Below is the link to the electronic supplementary material.

Supplementary Material 1

## Data Availability

No datasets were generated or analysed during the current study.

## Abbreviations

DR: Diabetic Retinopathy
ICGA: Indocyanine Green Angiography
OCTA: Optical Coherence Tomography Angiography
PRISMA: Preferred Reporting Items for Systematic Reviews and Meta-analyses
SS-OCTA: Swept-Source Optical Coherence Tomography Angiography
SD-OCTA: Spectral-Domain Optical Coherence Tomography Angiography
RPE: Retinal Pigment Epithelium
NDR: Diabetic Eyes without Diabetic Retinopathy
CVI: Choroid Vascularity Index
DCP: Deep Capillary Plexus
SCP: Superficial Capillary Plexus
NPDR: Non-proliferative Diabetic Retinopathy
PDR: Proliferative Diabetic Retinopathy
ETDRS: Early Treatment Diabetic Retinopathy Study
BCVA: Best-corrected Visual Acuity
HPi: Heterogeneity Packing Index
LDi: Linear Dispersion Index
EZ: Ellipsoid Zone
ERG: Electroretinogram
mfERG: Multifocal Electroretinogram
RAD: Response Amplitude Density
