# Supplementary material for: The role of optical coherence tomography angiography in assessing diabetic choroidopathy: a systematic review
Source: Int J Retina Vitreous. 2025 Jan 31;11:10. doi: 10.1186/s40942-024-00618-5 (PMC11786548; doi:10.1186/s40942-024-00618-5)
Supplement: Supplementary file 1 — Supplementary Material 1 [file 40942_2024_618_MOESM1_ESM.docx]

| **E-Table 1. Quality Assessment of Cohort Studies on the Role of Optical Coherence Angiography in Evaluating Diabetic Choroidopathy using Newcastle-Ottawa Scale** | | | | | | | | | |
| --- | --- | --- | --- | --- | --- | --- | --- | --- | --- |
| **First Author** | **Year** | **Representativeness of the exposed cohort** | **Selection of the non-exposed cohort** | **Ascertainment of exposure** | **Demonstration that outcome of interest was not present at start** | **Comparability of cohorts on the basis of the design/analysis** | **Assessment of outcome** | **Was follow-up long enough for outcomes to occur?** | **Adequacy of follow up of cohorts** |
| Fragiotta et al. [28] | 2023 | * |  | * | * |  | * | * | * |
| Chen et al. [44] | 2023 | * |  | * | * |  | * | * | * |
| Guo et al. [45] | 2023 | * |  | * | * |  | * | * | * |
| Wang et al.[40] | 2022 | * |  | * |  |  | * | * |  |

| **E-Table 2. Quality Assessment of Case Control Studies on the Role of Optical Coherence Angiography in Evaluating Diabetic Choroidopathy using Newcastle-Ottawa Scale** | | | | | | | | | |
| --- | --- | --- | --- | --- | --- | --- | --- | --- | --- |
| **First Author** | **Year** | **Selection** | | | | **Comparability** | **Exposure** | | |
|  |  | **Is the case definition adequate?** | **Representative of the cases** | **Selection of controls** | **Definition of controls** | **Comparability of cases and controls on the basis of design or analysis** | **Ascertainment of exposures** | **Same method of Ascertainment for cases and controls** | **Non-response rate** |
| **Bandello et al. [50]** | 2023 | * | * | * | * | ** | * | * | * |
| **Deng et al. [31]** | 2023 | * | * | * | * | ** | * | * | * |
| **Viggiano et al. [52]** | 2023 | * | * | * | * | ** | * | * | * |
| **Tan et al. [20]** | 2023 | * | * |  | * | ** | * | * | * |
| **Xiong et al. [17]** | 2022 | * | * | * | * | ** | * | * | * |
| **Liu et al. [41]** | 2022 | * | * |  | * | * | * | * | * |
| **Zhang et al. [10]** | 2021 | * | * |  | * | * | * | * | * |
| **Ryu et al. [39]** | 2021 | * | * |  | * | * | * | * | * |
| [**Zlatanović**](https://pubmed.ncbi.nlm.nih.gov/?sort=date&size=200&term=Zlatanovi%C4%87+M&cauthor_id=34853704) **et al. [16]** | 2021 | * | * |  | * | ** | * | * | * |
| **Stulova et al. [19]** | 2021 | * | * |  | * | * | * | * | * |
| **Agra et al. [27]** | 2021 | * | * |  | * | ** | * | * | * |
| **Loria et al. [15]** | 2021 | * | * |  | * | * | * | * | * |
| **Ro-Mase et al. [51]** | 2020 | * | * |  | * | ** | * | * | * |
| **Dai et al. [1]** | 2020 | * | * |  | * | ** | * | * | * |
| **Saif et al. [9]** | 2020 | * | * |  | * | * | * | * | * |
| **Dai et al. [6]** | 2020 | * | * |  | * | ** | * | * | * |
| **Lupidi et al. [37]** | 2020 | * | * |  |  | ** | * | * | * |
| **Borrelli et al. [54]** | 2020 | * | * |  | * | * | * | * | * |
| **Forte et al. [14]** | 2020 | * | * |  | * | * | * | * | * |
| **Sacconi et al. [23]** | 2019 | * | * | * | * | ** | * | * | * |
| **Mastropasqua et al. [46]** | 2019 | * | * | * | * | * | * | * | * |
| **Yang et al. [4]** | 2019 | * | * | * | * | * | * | * | * |
| **Yang et al. [38]** | 2019 | * | * | * | * | * | * | * | * |
| **Conti et al. [26]** | 2019 | * | * |  | * | * | * | * | * |
| **Li et al. [48]** | 2019 | * | * |  | * | ** | * | * | * |
| **Li et al. [18]** | 2018 | * | * |  | * | * | * | * | * |
| **Cao et al. [56]** | 2018 | * | * |  | * | * | * | * | * |
| **Carnevali et al. [24]** | 2017 | * | * |  | * | * | * | * | * |
| **Dimitrova et al. [25]** | 2017 | * | * | * | * | ** | * | * | * |
| **Choi et al. [5]** | 2017 | * | * | * | * | * | * | * | * |
